# Why water, sanitation and hygiene matter

**Published:** 2013

**Authors:** Georgia B Savage, Alison K Macintyre, James H Wicken, Yael Velleman, Virginia Sarah

**Affiliations:** WaterAid Australia, Melbourne, Australia.; WaterAid, London, United Kingdom.; Fred Hollows Foundation, London, United Kingdom.

Water, sanitation and hygiene (WASH) are crucial but often underplayed parts of the prevention and control of a number of neglected tropical diseases (NTDs).

Access to safe water and adequate sanitation, together with good hygiene practices, can reduce the transmission of some NTDs, for example trachoma and intestinal worms (page 29). Trachoma is transmitted by flies, fomites (e.g. skin, hair, clothing, or bedding) and direct contact. Preventing transmission of trachoma can be achieved through access to clean water, appropriate hygiene practices that promote face washing, and access to proper sanitation for the disposal of human waste. Intestinal worms, which affect nearly 900 million people worldwide, is most prevalent in communities where people have inadequate access to toilets and/or hand washing facilities. Worms are transmitted through faecal-oral contact or enter through the skin of the feet in areas of open defecation. Access to safe water and adequate sanitation will help communities affected by both trachoma and soil-transmitted helminths (STH) to escape from the perpetual cycle of infection and reinfection.

Some global^1^ and disease specific^2^ strategies have integrated WASH interventions into their programming guidelines. In the case of trachoma, for example, the inclusion of the T’ (face washing) and ‘E’ (environmental improvement) in the SAFE strategy formally acknowledges the strategic importance of incorporating WASH interventions for disease elimination.

## Some practical opportunities for integration

The acknowledgment of the importance of WASH for comprehensive NTD control has not always translated into effective incorporation of WASH interventions in NTD control programmes. Reasons for insufficient integration include the lack of awareness and information sharing between the WASH and NTD sectors, and a short-term view of disease control which fails to recognise, and invest in, the necessary long-term comprehensive activities required for sustainable WASH implementation.

People involved in WASH and NTD programmes should work closely together, in a coordinated manner. This might involve forming local and global partnerships, sharing information and research about disease impact, combining efforts when advocating for resources and political commitment to action, and planning sustainable programmes that meet goals for both the elimination of NTDs and the provision of adequate water, sanitation, and hygiene.

Unless WASH issues are adequately addressed, neglected tropical diseases will not be eliminated in the long term. Control may be achieved by the year 2020, but to prevent continued transmission and re-infection, sustainable WASH interventions are a necessity.

**Figure:**
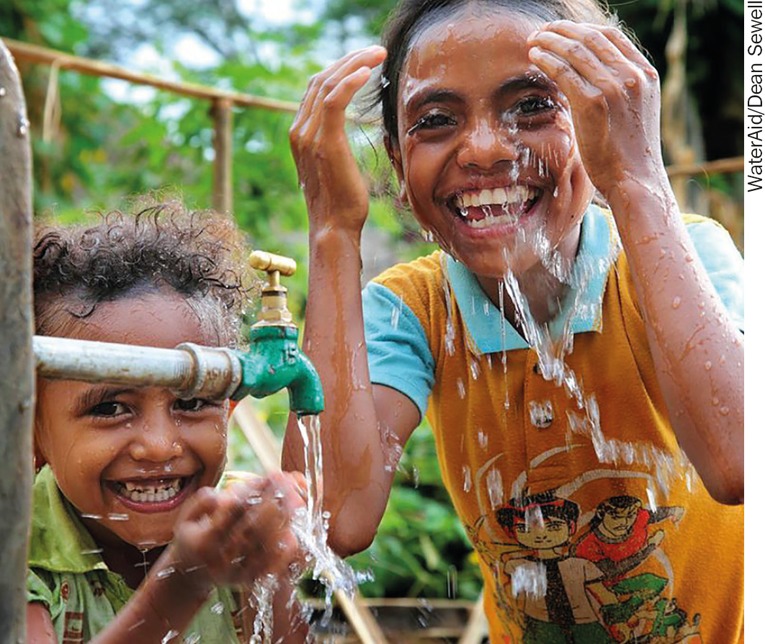
Access to water can help to combat NTDs.

Adapted from Savage G, Velleman X Wicken J, NTD NGO Network. WASH: The silent weapon against NTDs: working together to achieve prevention, control and elimination. Australia: WaterAid Australia; 2012.
